# Clinical and genetic analysis of two patients with primary ciliary dyskinesia caused by a novel variant of DNAAF2

**DOI:** 10.1186/s12887-023-04185-w

**Published:** 2023-12-05

**Authors:** Lili Dong, Lei Zhang, Xiao Li, Shiyue Mei, Yuelin Shen, Libing Fu, Shunying Zhao, Xiaolei Tang, Yu Tang

**Affiliations:** 1 Department of Respiratory Medicine, Children’s Hospital Affiliated to Zhengzhou University, Henan Children’s Hospital, Zhengzhou Children’s Hospital, Zhengzhou, China; 2 Henan Provincial Key Laboratory of Children’s Genetics and Metabolic Diseases, Children’s Hospital Affiliated to Zhengzhou University, Henan Children’s Hospital, Zhengzhou Children’s Hospital, Zhengzhou, China; 3 Department of Respiratory Medicine, Beijing Children’s Hospital, Capital Medical University, National Center for Children’s Health, Beijing, China; 4 Department of Pathology, Beijing Children’s Hospital, Capital Medical University, National Center for Children’s Health, Beijing, China

**Keywords:** Primary ciliary dyskinesia, Dynein axonemal assembly factor, Genotype, Variant, Chinese

## Abstract

**Background:**

The study describes the clinical manifestations and variant screening of two Chinese siblings with primary ciliary dyskinesia (PCD). They carry the same DNAAF2 genotype, which is an extremely rare PCD genotype in the Chinese population. In addition, the study illustrated an overview of published variants on DNAAF2 to date.

**Methods:**

A two-child family was recruited for the study. Clinical manifestations, laboratory tests, bronchoscopic and otoscopic images, and radiographic data were collected. Whole blood was collected from siblings and their parents for whole-exome sequencing (WES) and Sanger sequencing to screen variants.

**Results:**

The two siblings exhibited typical clinical manifestations of PCD. Two compound heterozygous variants in DNAAF2 were detected in both by WES. Nonsense variant c.156 C>A and frameshift variant c.177_178insA, which was a novel variant.

**Conclusion:**

The study identified a novel variant of DNAAF2 in Chinese children with a typical phenotype of PCD, which may enrich our knowledge of the clinical, diagnostic and genetic information of DNAAF2-induced PCD in children.

**Supplementary Information:**

The online version contains supplementary material available at 10.1186/s12887-023-04185-w.

## Background

According to European Respiratory Society (ERS) and American Thoracic Society (ATS), PCD is a rare inherited autosomal recessive disease caused by impaired function of the motile cilia, with an estimated incidence of one in 10,000–20,000 live births worldwide [1], resulting in recurrent respiratory inflammation, bronchiectasis, sinusitis, otitis media, and neonatal respiratory distress [2, 3]. Due to the random asymmetry of the left-right bodies, up to about 50% of PCD patients have situs inversus or ambiguous [4]. In addition to the clinical features, nasal nitric oxide (nNO) concentration, high speed video microscopy analysis (HSVMA) of cilia beat frequency and pattern, ciliary ultrastructure in cross section through transmission electron microscopy (TEM) are all helpful for the diagnosis of PCD, thereinto, genetic testing can help to confirm the diagnosis of PCD [2, 3].

The pathogenesis of PCD is cilia motility dysfunction. Movements of cilia can help to clear mucous from the airway, the flow of cerebrospinal fluid along the brain ventricular system, to support the transport of the oocyte to the uterus, and the movement of male germ cells along the female reproductive tract [5]. The normal structure of the cilia consists of the conserved basic 9 + 2 microtubule-based axonemes and several other functional modules [6]. Members of the multi-subunit motor protein complexes, outer dynein arms (ODAs) and the inner dynein arms (IDAs) are responsible for generating and regulating ciliary beating. Dynein arms are ATPase-based protein-complexes, hydrolyze ATP through dynein heavy chain, allowing the paired microtubules to slide relative to each other, leading to cilia curve over deformed microtubules [7, 8]. During the process, IDA causes the curvature of cilia, and ODA accelerates the active sliding of outer microtubules, leading to the cilia to generate effective propulsion, effectively paddling forward and backward. Appropriate rheological properties and functional structure of cilia are the basis for maintaining mucociliary clearance, which is essential for clearance defense of the respiratory tract [9].

There are two different types of respiratory cilia ODA, proximal A-tubule (type 1) and distal A-tubule (type 2). The components of ODA are gradually assembled into large multiprotein complexes by dynein axonemal assembly factor (DNAAFs) in the cytoplasm. DNAAF2, a facilitator of dynein pre-assembly, also known as Ktu/PF13, has been reported to encode the cytoplasmic proteins responsible for ODA type 2 and IDA light DNALI1 from ciliary axonemes. Variants in DNAAF2 lead to loss of cilia motility and PCD [5, 6].

In this paper, we reported the clinical manifestations, diagnosis and treatment processes of two siblings with PCD caused by the same compound heterozygous variants in DNAAF2, c.156 C>A /c.177_178insA, an extremely rare PCD genotype in Chinese population, which had never discovered in children. Furthermore, c.177_178insA gene variant is considered as a novel variant.

## Methods

### Patients

The proband, a 7-year-old girl (II:1) and a 10-month-boy (II:2), were both born at full term, without remarkable family histories. Neither of the two probands had any history of fatty diarrhea, malnutrition, meconium intestinal obstruction. Both had normal cellular and humoral immune function, normal blood amylase and lipase levels, and negative pulmonary tuberculosis infection test. Clinical manifestations, laboratory tests, bronchoscopic and otoscopic images, and radiographic data were collected. Respiratory pathogens were identified from cultured bronchoalveolar lavage specimens. Whole blood was collected from siblings (II:1 & II:2) and their parents for whole-exome sequencing (WES) and Sanger sequencing. Literature of all DNAAF2 variants since the first reported were also searched. The clinical data of these patients were summarized. All methods were carried out in accordance with relevant guidelines and regulations. Informed consent was obtained from the parents. This study was conducted in accordance with the revised Declaration of Helsinki and approved by Ethics Committee of Henan Children’s Hospital (Approved No. 2022-k-049).

### NNO measurement

Nasal NO was measured during quiet exhalation with EcoMedics CLD 88 ANALYZER (Dürnten, Switzerland). nNO measurement of proband II:1 was in line with ATS/ERS recommendations [2, 3]. For proband II:2, NO values were tested in both nostrils for 60s during tidal breathing and the larger value was recorded. nNO production (nL/min) was calculated by multiplying the nNO concentration (ppb) by the sampling flow rate (0.3 L/min).

### Transmission electron microscopy

Obtained by bronchial biopsy, bronchial mucosa biopsy specimens were immediately immersed in glutaraldehyde at 4 °C, washed overnight, fixed in 1% osmium tetroxide, dehydrated, and then embedded in epoxy resin. After polymerization, sample sections were placed onto copper grids and stained with aqueous uranyl acetate and Reynold’s lead citrate. Ciliary ultrastructure was analyzed with a transmission electron microscope (JEM-1400; Jeol, Tokyo, Japan).

### Genetic sequencing

Genomic DNA samples were isolated from EDTA-treated peripheral blood of probands and their parents using Chemagic 360 kit (CMG-536, PerkinElmer, USA). With standard protocols, whole exome sequencing was performed at WE-HEALTH Biomedical Technology Company (Shanghai, China) and raw data was assessed for quality and sequenced using the Illumina HiSeq X System sequencer after enriched. Sequence read was aligned to the NCBI human reference genome (hg19) and variants were evaluated according to American College of Medical Genetics and Genomics (ACMG) classification criteria. Sanger-sequencing was then used to confirm the genetic variation.

## Results

**Fig. 1:**
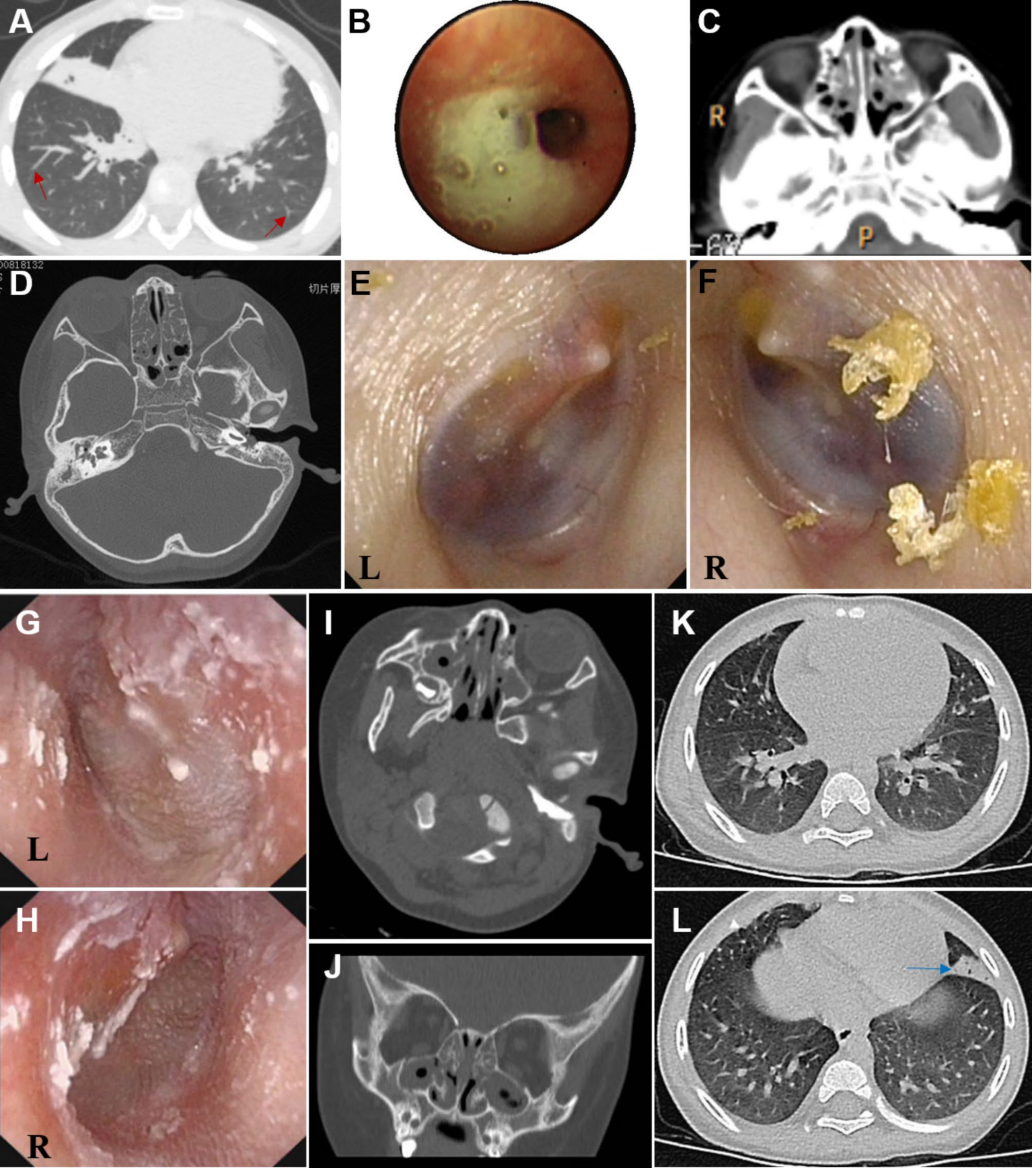
Clinical features of the probands, images A-F were of II:1 and G-L were of II:2. The chest high-resolution computed tomography (HRCT) scan showed bronchiectasis (red arrows) and atelectasis in the right middle lobe **(A)**, sinusitis **(C)** and otitis media **(D)**. Bronchoscopy revealed a large amount of mucilage secretions in the right middle lobe. Otoscope findings of otitis media in left ear **(E)** and the right one **(F)**. Otoscope findings of otitis media of left and right ears (G&H). The CT scan of nasopharynx showed sinusitis (I&J). Chest HRCT scan illustrated atelectasis in lingular lobe (blue arrow, K&L)

The proband II:1 presented with wet cough and running nose for 2 months. She was diagnosed with pneumonia and treated in a local hospital for 15 days, however, the symptoms were not alleviated. Computed tomography (CT) (Fig. 1A, C) scanning of her lungs and sinuses showed atelectasis and potential bronchiectasis in the right middle lobe, and sinusitis with hypertrophy of tonsils and adenoids. Before being transferred to the respiratory medicine department of our hospital, she had suffered from recurrent respiratory tract infection for almost 5 years. Physical examination found pharyngeal congestion and tonsil swelling, with normal growth and development. Her nNO concentration (11.55nL/min) was well below the PCD-specific nNO cutoff value (77nL/min) [2, 3]. The pulmonary function tests were normal. Bronchoscopy revealed a large amount of mucilage secretions in lingula segments, left upper and right middle lobes (Fig. 1B). Bronchoalveolar lavage (BAL) culture showed that *Haemophilus infuenzae (H. influenzae)* was positive. Pure-tone audiometry confirmed conductive hearing loss. The air-bone gap of the right ear was10dB to 25dB, and that of the left ear was 15dB to 35dB. Otoscope and inner ear CT indicated otitis media in both of her ears, which was responsible for the hearing loss (Fig. 1D, E, F).

Azithromycin plays an anti-inflammatory role in the treatment of *H. influenzae*. Budesonide and inhaled acetylcysteine were used for anti-inflammation and sputum dilution. In terms of pulmonary rehabilitation, mechanical vibration sputum expectoration, postural drainage and effective breathing techniques were performed to accelerate the clearance of lung mucus. During follow-up, she had improved symptoms but persistent atelectasis in the right middle lung (shown in supplemental Fig. 1) and recurrent sinusitis and otitis media (shown in supplemental Fig. 2).

The proband II:2 presented with cough and runny nose for one month. Actually, the runny nose initially started at the age of 2 months. He was diagnosed as otitis media (Fig. 1G&H), sinusitis (Fig. 1I&J), pneumonia in both lungs and atelectasis in lingular lobe (Fig. 1K&L), and hypertrophy of adenoids. Physical examination by stethoscope found crackles, and his growth and development were normal. Pure-tone audiometry confirmed conductive hearing loss. The air-bone gap of the right ear was 40dB to 60dB, and that of the left ear was 30dB to 55dB. Bilateral tympanotomy with tube placement and adenotomy were performed. His nNO was 9.84nL/min, even lower than that of proband II:1, which might be related to the recurrent respiratory infection. Pulmonary function tests showed obstructive ventilatory dysfunction. By transmission electron microscopy (TEM), ODAs and IDAs of bronchial cilia were not clearly displayed, indicating the possibility of the defect of ODAs and IDAs (Fig. 4). In addition, the echocardiography revealed residual left superior vena cava. Due to a history of emesis, he was diagnosed as intestine malrotation by abdominal ultrasound at 1.5 years old.

**Fig. 2:**
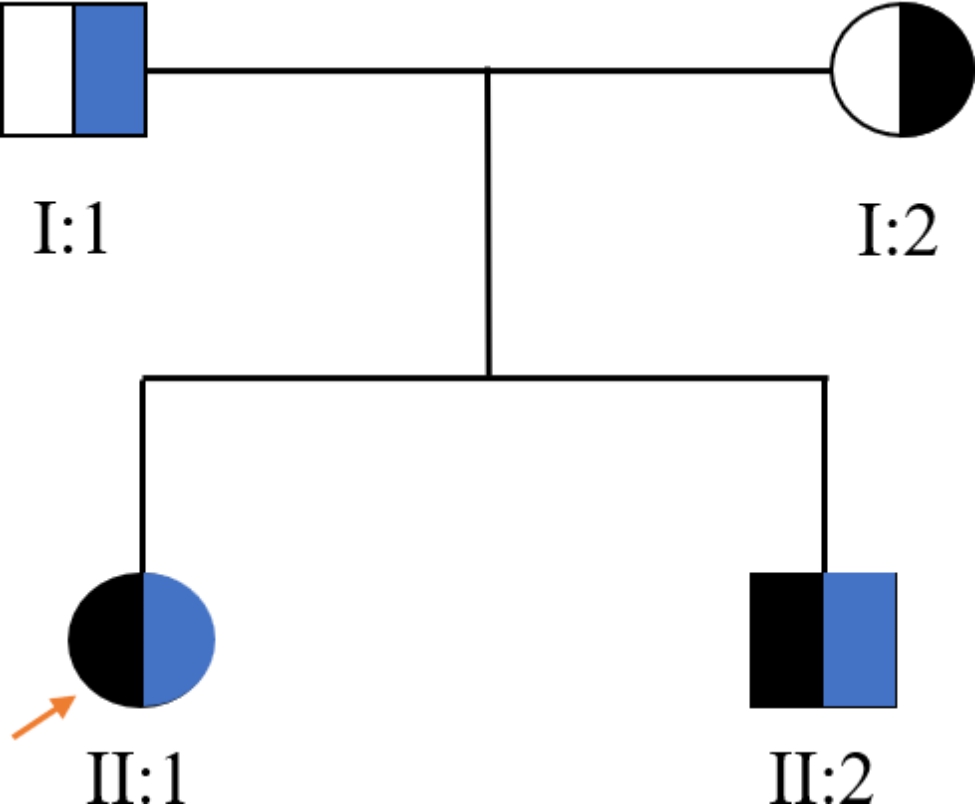
Pedigree of the family. Circles indicate females, squares indicate males, solid symbols indicate patients, arrow indicates the first diagnosed proband, blue indicates c.177_178insA (p.E60fs*3), black indicates c.156 C > A (p.Y52*)

**Fig. 3:**
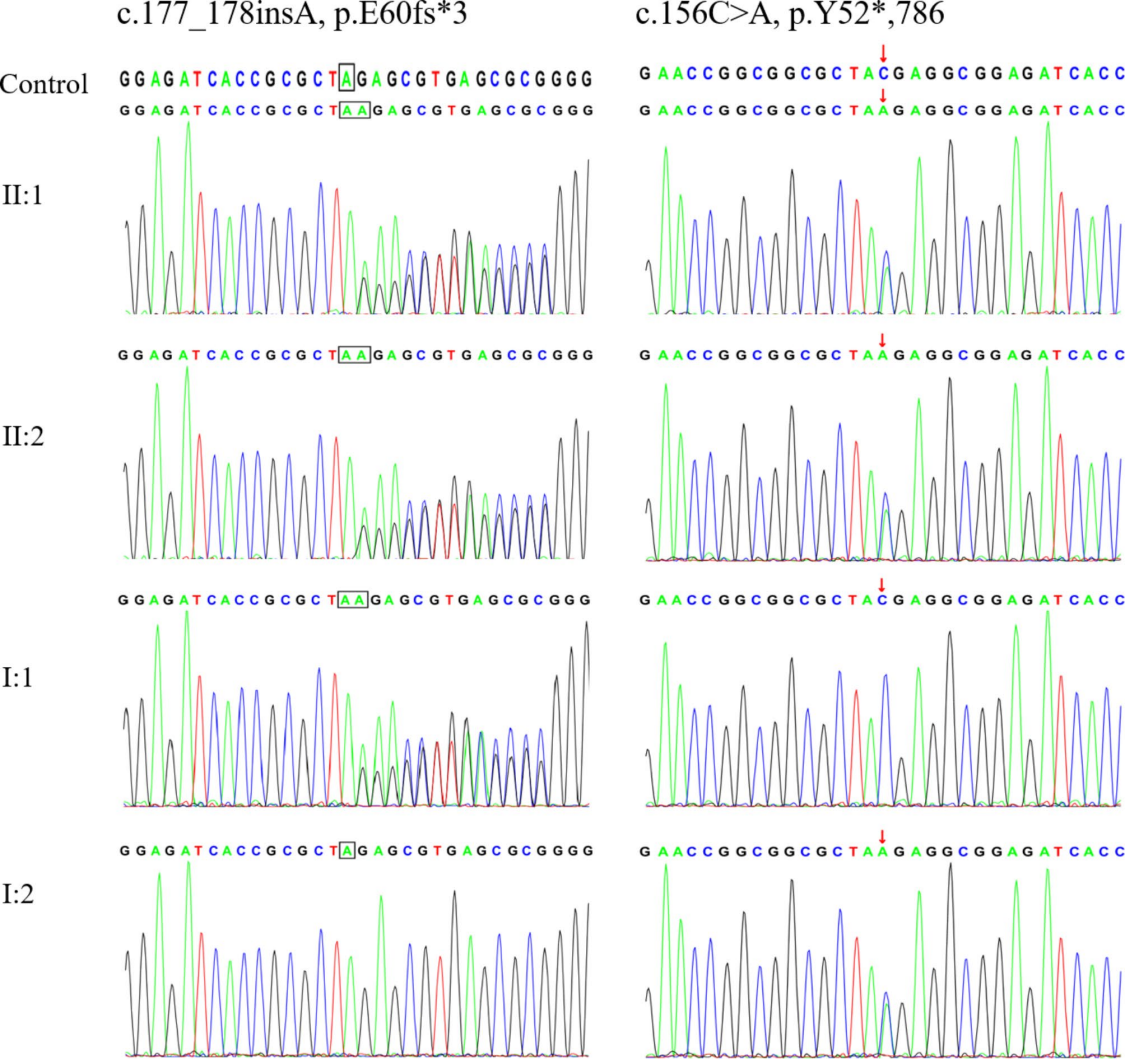
Sanger DNA sequencing chromatogram of probands and their parents. A novel variant DNAAF2, c.177_178insA (p.E60fs*3) was identified in the probands, their father had a heterozygous variant at the same location, whereas their mother did not. Another variant DNAAF2, c.156 C > A (p.Y52*,786) was also identified in both probands, their mother had a heterozygous variant at the same location, whereas their father did not

**Fig. 4:**
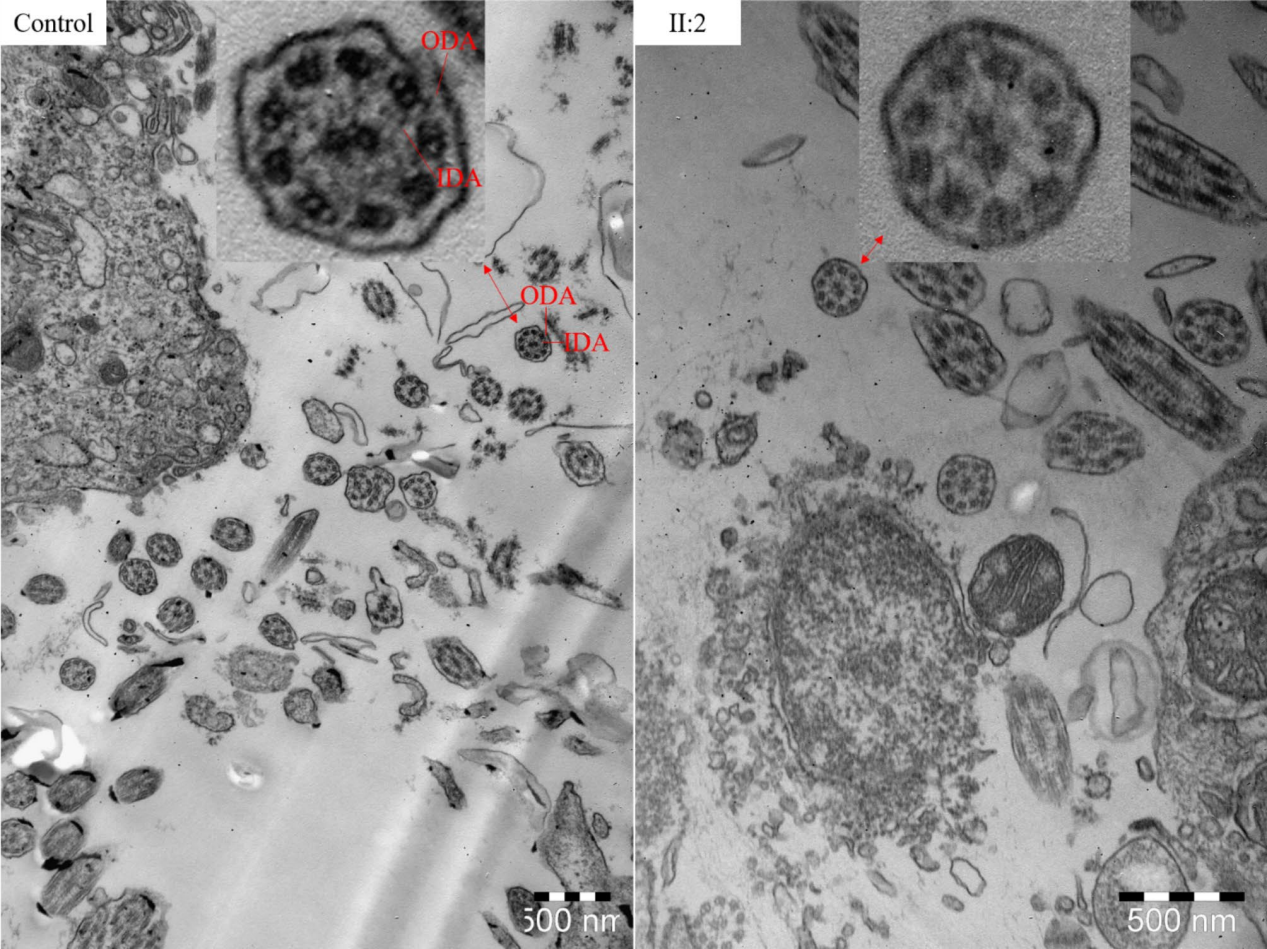
TEM analysis of bronchial mucosal biopsy specimens displayed loss of ODAs and IDAs

After treated with azithromycin, budesonide inhalation solution, and adjuvant mechanical vibration sputum expectoration, he was discharged soon. Although his symptoms improved during the 2-year follow-up, he still suffered from recurrent respiratory tract infection for 6–7 times, and his HRCT and otitis media became worse (shown in supplemental Figs. 3&4).

With the informed consent of the child’s parents, 2ml whole blood was separately collected from the siblings and their parents for WES and Sanger sequencing. Two compound heterozygous variants in DNAAF2 were found in both siblings, one nonsense variant of NM_018139: c.156 C > A (p.Y52*) was inherited from the mother (I:2) and one frameshift variant NM_018139: c.177_178insA (p.E60fs*3) was inherited from the father (I:1, Figs. 2 and 3). The variant of c.177_178insA (p.E60fs*3) has not been reported before.

## Discussion

In this study, two compound heterozygous variants of DNAAF2 were identified in two siblings with similar PCD characteristics, including recurrent pneumonia dominated by the right middle and left lingular lobes with atelectasis, early bronchiectasis, sinusitis, otitis media, and significantly reduced nNO levels. One of the variants is the nonsense variant of NM_018139: c.156 C > A (p.Y52*), which has already been reported in a Chinese family and proved to be pathogenic [10]. Another variant is the frameshift variant (NM_018139: c.177_178insA (p.E60fs*3), which is a novel variant.

**Fig. 5:**
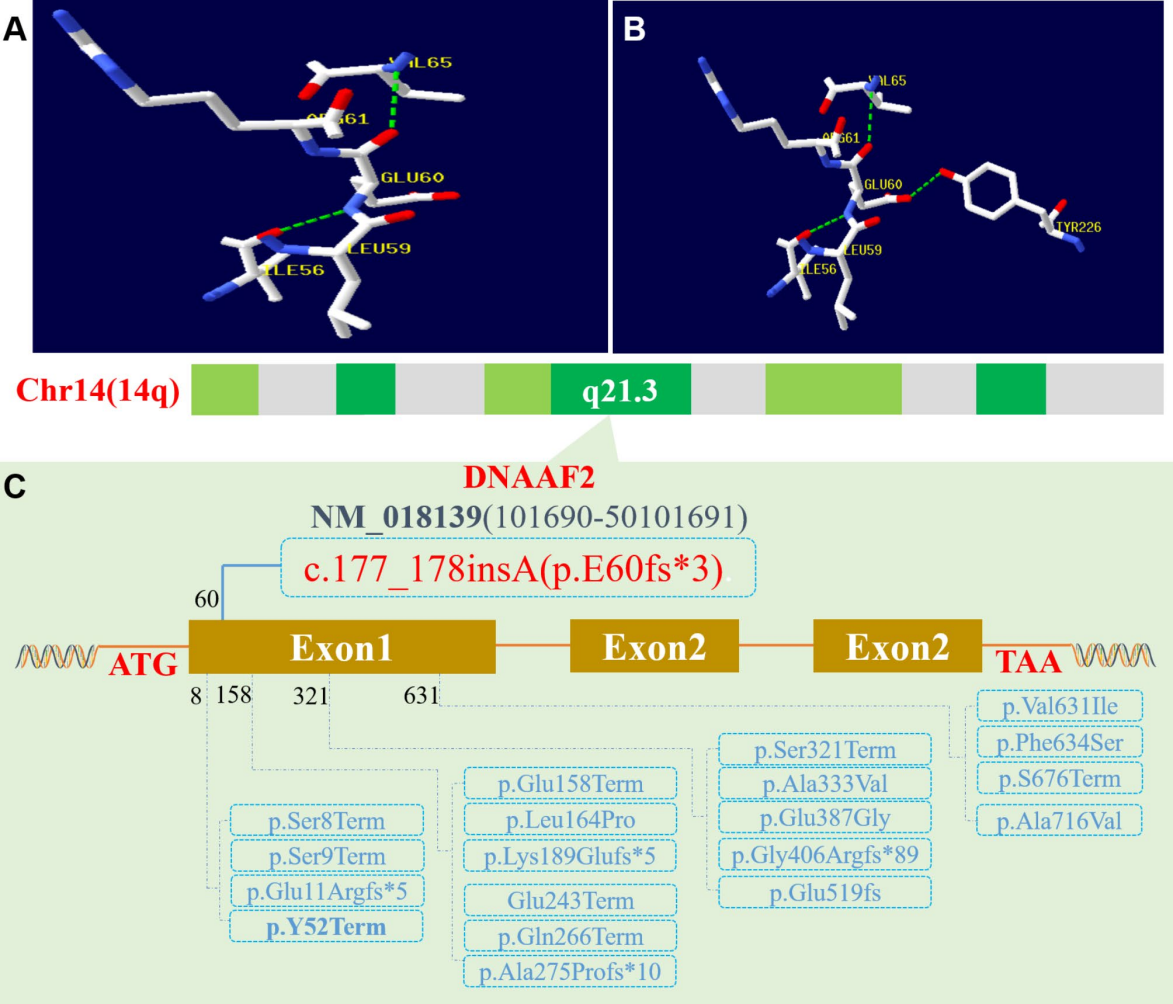
Variants of DNAAF2 gene identified in the probands and all published variants on protein level. The newly identified c.177_178insA (p.E60fs*3) variant structure **(A)** and its wide type **(B)**, and the protein in bold **(C)** was another identified variant in this study and other published variants on protein level were all displayed **(C)**

DNAAF2 is located on chromosome 14q21.3 and consists of three exons encoding cDNAs [10]. The variant p.E60fs*3 is located in exon1, near another variant p.Y52*. Exon1 is consists of 621 amino acids, and E60fs*3 variant leads to early transcription termination after 63 amino acids (Fig. 5). According to the standards and guidelines of American College of Medical Genetics and Genomics (ACMG), the frameshift variant p.E60fs*3 variant was pathogenic. In addition, none of cystic fibrosis (CF) or primary immune deficiency (PID) related genes were detected, which may also lead to recurrent pneumonia, atelectasis and bronchiectasis. Based on the clinical manifestations and hereditary mode of compound heterozygous variants from both parents, it is reasonable to consider that the novel p.E60fs*3 variant of DNAAF2 is responsible for the pathogenesis of PCD.

Over 50 genes have been reported to be associated with PCD, including DNAH5, DNAH11, DNAI1, DNAI2, DNAL1, TXNDC3, DNAAF1, DNAAF2, etc. [1, 6, 11]. In 2008, two heterozygous variants c.C23A [pS8X] and c.1214-1215insACGATACCTGCGTGGC [p.G406Rfs89X] variants of DNAAF2 were first identified in two consanguineous families with PCD presented with recurrent respiratory tract infections, laterality defects and impaired fertility [6]. DNAAF2 functions as a facilitator of dynein pre-assembly. The dysfunction of DNAAF2 is associated with the specific defects of the interaction between intermediate and heavy chains in the cytoplasm, leading to complete or partial loss of ODAs and IDAs and loss of motility [6]. Since then, only 14 PCD cases have been reported to be caused by DNAAF2 with 17 variants. The cases of DNAAF2 variant are summarized in Table 1 and variants on protein level are illustrated in Fig. 5 [6, 10, 12–24]. DNAAF2 gene is rare for Chinese Children. As reported by Guan et al. in 81 Chinese children, the genes causing the highest variant rate of PCD was DNAH11, followed by HYDIN, DNAH5, CCDC39, DNAH1 and CCNO, no variant of DNAAF2 gene was detected [25]. In Chinese adult patients, Sun et.at have identified two heterozygous variants, c.C156A [p.Y52X] and c.C26A [p.S9X], in the DNAAF2 gene, leading to defects in the outer dynein arms and inner dynein arms, thereby resulting in PCD with the manifestation of male infertility [10]. Lu C et al. detected another two heterozygous variants, c.491T > C [p.L164P] and c.822del [p.A275Profs*10], in two females with bronchiectasis, sinusitis, situs inversus, and infertility. In addition, one woman with c.491T > C variant had scoliosis [24].

**Table 1:** Cases of published DNAAF2 variants. RDS, respiratory distress syndrome; TEM, transmission electron microscopy; VSD, ventriculap septal defect; IDA, inner dynein arm; ODA, outer dynein arm; - not available

cDNA change (Protein change)	Sex	Age( years)	Ethnicity	Clinical features	Ultrastructural defect
c.1160 A > G(p. Glu387Gly)	F	-	Turkish	-	-
c.472G > T(p.Glu158Term)	F	7	-	VSD, Eisenmenger syndrome	-
c.23 C > A(p.Ser8Term)	M	-	-	Chronic otitis media and sinusitis, recurrent pneumonia, bronchiectasis, removed middle lobe, complete situs inversus, impaired fertility	ODA + IDA
c.1199_1214dupACGATACCTGCGTGGC (p.Gly406Argfs*89)	F	-	-	Chronic otitis media and sinusitis, recurrent pneumonia, bronchiectasis, complete situs inversus	ODA + IDA
c.564dupG(p.Lys189Glufs*5)	-	-	-	-	ODA + IDA
c.2147 C > T(p. Ala716Val)	F	-	European	Developmental disorder	-
c.156 C > A(p.Tyr52Term)	M	40	Asian	Recurrent bronchitis and pneumonia, chronic sinusitis, bronchiectasis, situs inversus, infertility	ODA + IDA
c.26 C > A(p.Ser9Term)					
c.727G > T(Glu243Term)	-	-	Swedish	-	-
c.2027_2028delCT(p.S676Term)	-	-	-	-	-
c.962 C > A(p.Ser321Term)	M	-	Greek-Cypriot	-	-
c.998 C > T(p.Ala333Val)	-	-	Dutch	-	ODA + partial IDA
c.1901T > C(p.Phe634Ser)					
c.796 C > T(p.Gln266Term)	M	30	Asian	Kartagener Syndrome, productive cough, hemoptysis, nasosinusitis, left-right laterality defects, azoospermia	-
c.1555delG(p.Glu519fs)	M	-	-	-	-
c.1891G > A(p.Val631Ile)	M	-	-	Infertility	-
c.31delG(p.Glu11Argfs*5)	F	14	Italian	Neonatal RDS, bronchiectasis, sinusitis	ODA + IDA
c.491T > C (p.Leu164Pro)	F	26	Asian	Scoliosis, bronchiectasis, sinusitis, situs inversus and infertility	ODA
c.822del (p.Ala275Profs*10)	F	53	Asian	Bronchiectasis, sinusitis and infertility	-
c.177_178insA (p.E60fs*3) Novel	F	7	Asian	Recurrent respiratory tract infection, atelectasis, bronchiectasis, sinusitis and otitis media	-
	M	10/12	Asian	Pneumonia, atelectasis in lingular lobe, sinusitis, otitis media and hypertrophy of adenoids	ODA + IDA

Consequently, we made an early diagnosis of PCD in two siblings with a rare novel variant of DNAAF2 gene. Early PCD may be easily misdiagnosed as common pneumonia or sinusitis. PCD should be considered when a child presented with recurrent pneumonia or atelectasis accompanied with recurrent sinusitis or otitis media. The characteristic HRCT manifestations include right middle and left lingular atelectasis, thickened bronchial wall, and mild bronchiectasis, which can provide a diagnostic clue for PCD. Moreover, the decrease of nNO is conducive to the further diagnosis, and genetic tests is conducive to confirming the diagnosis. TEM of bronchial cilia is also helpful for the diagnosis of PCD.

## Conclusion

Our study suggested that, c.156 C>A/c.177_178insA variant of DNAAF2 is a novel pathogenic genotype in Chinese children with typical clinical features of PCD, which may broaden the gene spectrum, and enrich our knowledge of the clinical, diagnostic and genetic information of DNAAF2-induced PCD in children.

### Electronic supplementary material

Below is the link to the electronic supplementary material.

Supplementary Material 1

Supplementary Material 2

Supplementary Material 3

Supplementary Material 4

## Data Availability

The datasets generated and/or analyzed during the current study are available in the Genome Sequence Archive (Genomics, Proteomics & Bioinformatics 2021) in National Genomics Data Center (Nucleic Acids Res 2022), China National Center for Bioinformation / Beijing Institute of Genomics, Chinese Academy of Sciences (accession number: HRA002729) that are publicly accessible at https://ngdc.cncb.ac.cn/gsa-human.

## Abbreviations

PCD: Primary ciliary dyskinesia
WES: Whole-exome sequencing
ERS: European Respiratory Society
ATS: American Thoracic Society
HSVMA: High speed video microscopy analysis
TEM: Transmission electron microscopy
ODAs: Outer dynein arms
IDAs: Inner dynein arms
DNAAFs: Dynein axonemal assembly factor
nNO: Nasal nitric oxide
CT: Computed tomography
BAL: Bronchoalveolar lavage
HRCT: High-resolution computed tomography
ACMG: American College of Medical Genetics and Genomics
CF: Cystic fibrosis
PID: Primary immune deficiency
