# Supplementary figures and images for: Clinical and genetic analysis of two patients with primary ciliary dyskinesia caused by a novel variant of DNAAF2
Source: BMC Pediatr. 2023 Dec 5;23:616. doi: 10.1186/s12887-023-04185-w (PMC10696777; doi:10.1186/s12887-023-04185-w)

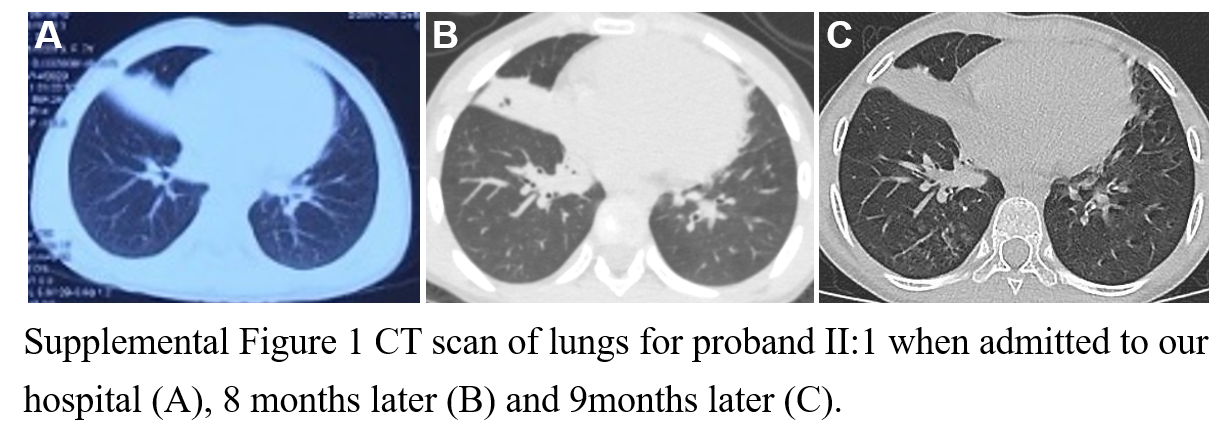

Supplement: Supplementary file 1 — Supplementary Material 1 [file 12887_2023_4185_MOESM1_ESM.png]

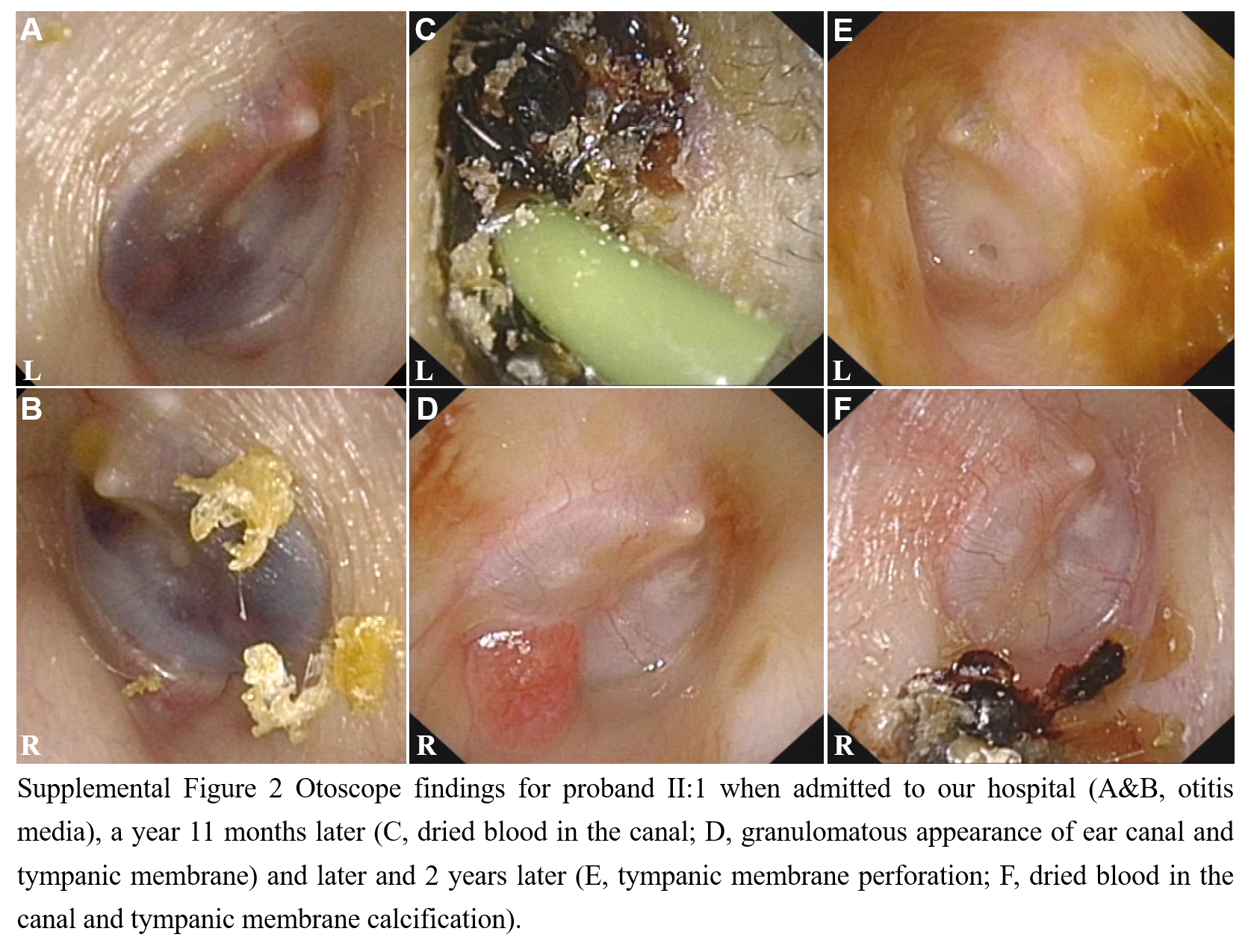

Supplement: Supplementary file 2 — Supplementary Material 2 [file 12887_2023_4185_MOESM2_ESM.png]

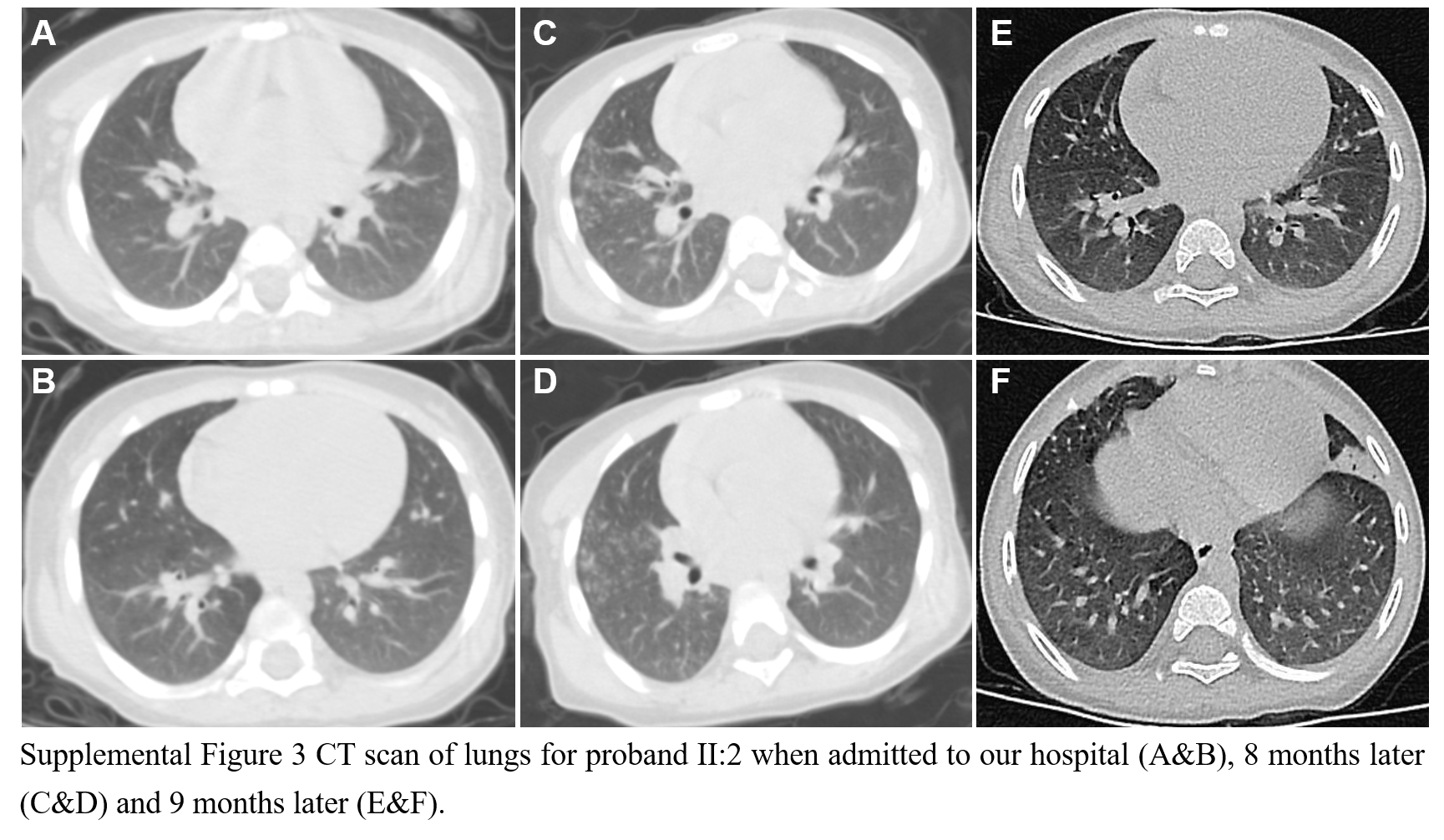

Supplement: Supplementary file 3 — Supplementary Material 3 [file 12887_2023_4185_MOESM3_ESM.png]

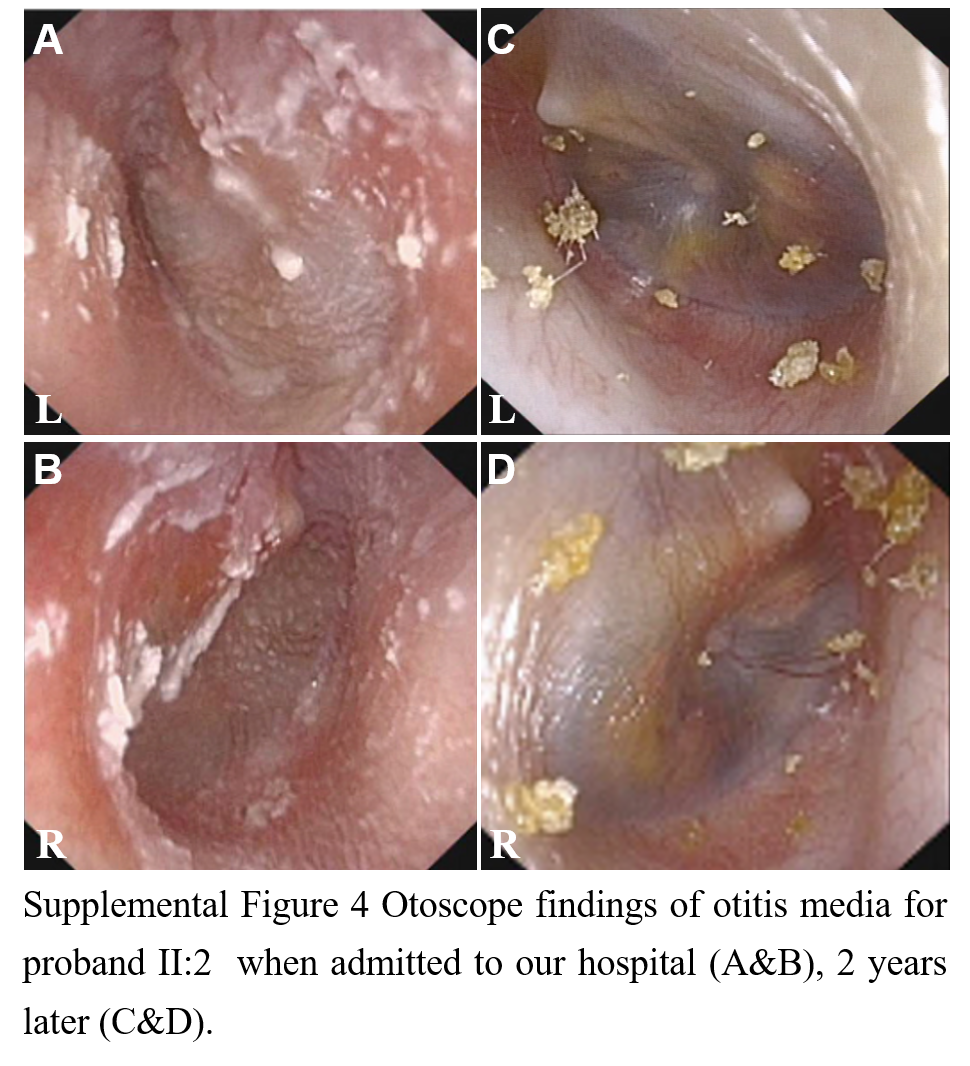

Supplement: Supplementary file 4 — Supplementary Material 4 [file 12887_2023_4185_MOESM4_ESM.png]
